# Construction and application of a medication adherence management program based on TIR for patients with ischemic stroke

**DOI:** 10.3389/fpubh.2026.1869629

**Published:** 2026-07-24

**Authors:** Jie Dong, Yayan Yi, Yimei Hu, Jinying Jiang, Guohua Zhao

**Affiliations:** 1 Department of Nursing, The Fourth Affiliated Hospital of School of Medicine, and International School of Medicine, International Institutes of Medicine, Zhejiang University, Yiwu, China; 2 Department of Neurology, The Fourth Affiliated Hospital, and International School of Medicine, Zhejiang University School of Medicine, Zhejiang University, Yiwu, China

**Keywords:** ischemic stroke, medication adherence, medication beliefs, medication management, timing it right theory

## Abstract

**Objective:**

This study aimed to construct a multidisciplinary medication management program based on the Timing It Right Theory (TIR) and evaluate its effects on medication adherence, medication beliefs, and medication knowledge in patients with ischemic stroke.

**Methods:**

A total of 143 patients with ischemic stroke from a tertiary hospital in Zhejiang from January to October 2024 were enrolled in the study. To avoid cross-contamination, patients were assigned to an intervention group (*n* = 70) and a control group (*n* = 73) based on their hospital floor. The research team systematically searched relevant Chinese and English databases and developed a TIR-based Medication Management Program for patients with ischemic stroke. The control group received routine medication guidance and discharge education. In addition to routine care, the intervention group received TIR-based medication management from admission to 3 months after discharge. Medication knowledge was assessed using Medication Knowledge Questionnaire at baseline and discharge. Medication adherence and medication beliefs were assessed using the 8-item Morisky Medication Adherence Scale and the Beliefs about Medicines Questionnaire-Specific at baseline, discharge, and 1 and 3 months after discharge. The trends and differences between the two groups were analyzed.

**Results:**

Non-significant differences were found in baseline scores of medication knowledge, medication adherence, or medication beliefs between the two groups (*p* > 0.05). At discharge, the medication knowledge score in the intervention group (38.21 ± 6.02) was significantly higher compared to that in the control group (34.62 ± 4.98) (*p* < 0.05). Repeated measures analysis of variance demonstrated significant differences in medication adherence and medication belief scores between the two groups at discharge, 1 month after discharge, and 3 months after discharge (*p* < 0.05). Medication adherence scores in the intervention group were higher than those in the control group at all follow-up time points. In the Beliefs about Medicines Questionnaire, the intervention group had higher necessity scores and lower concerns scores than the control group, and the differences were statistically significant (*p* < 0.05).

**Conclusion:**

The multidisciplinary medication management program assigned to TIR can effectively improve medication knowledge, optimize medication beliefs, and enhance medication adherence in patients with ischemic stroke.

## 1 Introduction

Stroke is a common cerebrovascular disease characterized by high incidence, high disability, high mortality, a heavy disease burden, multiple comorbidities, and a steadily increasing recurrence rate (1). It has become the second leading cause of mortality and the third leading cause of death and disability combined globally, making it an urgent public health concern (2, 3). Ischemic stroke is the most common subtype, accounting for approximately 77.8% of all stroke cases (2). Recurrence is a critical factor that further increases the burden of stroke. A global systematic review and meta-analysis published in JAMA in 2025 reported that the five-year risk of stroke recurrence in patients with transient ischemic attack or minor stroke was 12.8%, rising to 19.8% at 10 years, with approximately 10% of recurrent strokes being fatal (4). Studies conducted in Chinese populations have further confirmed that recurrent stroke is often associated with more severe functional disability and poor health outcomes (5).

Medication adherence is defined as the extent to which patients take their medications as prescribed by healthcare providers, including dosage, frequency, and timing (6). One research has indicated that the standardized implementation and sustained adherence to effective secondary preventive pharmacotherapy can reduce the risk of stroke recurrence (7), which is crucial for lowering recurrence risk and managing stroke-related complications. Medication adherence is therefore the key to ensuring the effectiveness of secondary prevention measures (8). Despite the well-documented benefits of pharmacological therapy in lowering stroke incidence, recurrence, and adverse outcomes, adherence to secondary prevention medications among patients with ischemic stroke remains suboptimal and tends to decline over time, underscoring an urgent need for targeted interventions (8, 9). Medication adherence is a dynamic process influenced by multiple factors, including patients’ perception of their disease, health beliefs, social support, and medication-specific beliefs (10, 11). After hospital discharge, patients face distinct challenges at different stages of recovery (8). In the early post-discharge period, some patients may reduce or discontinue medication on their own because they perceive symptom improvement. During the chronic disease management stage, cognitive and behavioral barriers gradually become prominent, including forgetting to take medication, difficulty adhering to complex treatment regimens, and concerns about potential adverse drug reactions. This suggests that medication adherence management requires stage-specific and individualized support strategies. Furthermore, current intervention studies have mainly focused on unimodal strategies, including standalone health education, post-discharge telephone follow-up, and information technology-based medication counseling (12). These interventions often fail to adequately address the distinct medication management challenges faced by patients and their primary caregivers at different stages of the disease trajectory, while also overlooking patients’ baseline cognitive levels related to medication adherence (12, 13). The Timing It Right Theory (TIR), introduced by Cameron and colleagues (14) in 2008, emerged from their investigation into the evolving needs of stroke caregivers and provides a conceptual framework to guide the delivery of timely, targeted support services to patients and their caregivers across diverse healthcare environments. TIR delineates the disease trajectory into five sequential stages: Diagnosis, stabilization, discharge preparation, implementation, and adaptation. A central tenet of the theory is that the type and intensity of interventions and support should be dynamically consistent with the evolving needs of patients and their caregivers at each distinct stage. TIR theory has demonstrated favorable effectiveness in home-based rehabilitation and caregiver support for stroke survivors (15, 16). Furthermore, the theory has gradually been extended to the management of other chronic diseases, such as diabetes mellitus and acute myocardial infarction (17), demonstrating promising application value (18).

Therefore, this study developed a medication management program grounded in TIR, shifting adherence interventions for stroke patients from generalized guidance toward precision support. By addressing the evolving medication management needs of patients and caregivers at each stage of the disease trajectory, the program enhances their understanding of and beliefs about medication adherence, reduces medication-related concerns, and ultimately promotes better medication-taking behaviors.

## 2 Materials and methods

### 2.1 Participants

This was a quasi-experimental study. Patients were recruited using a convenience sampling method from the neurology department of a tertiary hospital in Zhejiang Province between January and October 2024. To avoid cross-contamination, patients were allocated to the intervention group or the control group based on their hospital floor rather than by individual randomization. Although patients and caregivers were not blinded due to the nature of the intervention, outcome assessors remained blinded to group allocation.

A two-sided test was applied, with *α* = 0.05, t*α*/2 = 1.96, *β* = 0.01, and t*_β_* = 1.282. Based on a literature review, the standard deviation of medication adherence scores in patients with chronic diseases before and after intervention was 11.856, and the expected mean difference (*δ*) was 9.61 (19). The sample size was calculated using the formula for comparing two independent sample means:


N1=N2=2[s∗(tα/2+tβ/δ)]2

The calculation yielded a required sample size of 32 participants per group. With a 20% attrition rate, 40 participants per group were needed, yielding a total of 80 participants. Finally, 150 patients were included. The inclusion criteria were as follows: (1) Patients diagnosed with ischemic stroke according to the Chinese Guidelines for the Diagnosis and Treatment of Acute Ischemic Stroke 2018, confirmed by CT or MRI; (2) aged ≥ 18 years; (3) conscious and able to understand and respond to relevant questions; (4) willing to participate voluntarily and provide written informed consent; (5) taking at least one oral medication for at least 3 months prior to admission. The exclusion criteria included the following: (1) Concurrent severe organic damage to other organs, including severe cardiac, hepatic, or renal insufficiency, malignancy, or respiratory failure; (2) death during hospitalization; (3) presence of mental illness or uncorrected psychiatric and behavioral abnormalities; (4) no prescribed discharge medications. This study was approved by the hospital ethics committee (No.: 2024–1,028-4). All patients provided written informed consent before participation.

### 2.2 Intervention

#### 2.2.1 Control group

The control group received routine medication education and follow-up management. Upon admission, nurses assessed the patient’s medication history and completed and verified the current medication list. During hospitalization, nurses provided patients and their caregivers with educational materials on stroke-related medications. They delivered routine medication instructions, including verbal education on drug names, dosages, administration routes, and precautions. Prior to discharge, nurses conducted discharge counseling covering prescribed medications, post-discharge dietary and activity recommendations, and the schedule for subsequent follow-up visits. After discharge, follow-up nurses contacted patients through the follow-up information platform using text messages within 2 weeks. The follow-up assessed patients’ post-discharge functional recovery, blood pressure, and glucose monitoring status, missed medication, and adverse drug reactions. For patients with a history of hypertension and/or diabetes, the follow-up included a simple yes/no question regarding whether they had been monitoring their blood pressure or blood glucose as recommended. For patients who did not respond to text messages, telephone follow-up was conducted. Additionally, patients attended stroke clinic follow-up visits at 2 and 4 weeks after discharge. All control group patients received follow-up contacts at the same frequency and via the same format as the intervention group. However, the content of these follow-ups was limited to general health assessments and routine monitoring.

#### 2.2.2 Intervention group

##### 2.2.2.1 Establishment of the research team

The research team consisted of nine members: One neurologist, one head nurse, three neurological specialized nurses, one geriatric nursing specialist, one clinical pharmacist, one psychological counselor, and one rehabilitation therapist. The team was led by the head nurse of the cerebrovascular specialty ward, who coordinated the development and implementation of the medication service program based on TIR. The neurologist was responsible for assessing patients’ conditions, formulating treatment plans, and reviewing the safety and appropriateness of medication orders to prevent and manage related risks. Specialized nurses, in collaboration with the clinical pharmacist, psychological counselor, and rehabilitation therapist, jointly implemented the intervention measures. Additionally, the research nurse was responsible for conducting literature searches and collecting and organizing data throughout the intervention. The team adhered to a patient-centered, systematic, and collaborative working model to comprehensively address patients’ health needs at each stage of the disorder trajectory.

##### 2.2.2.2 Development of TIR-based medication management program for ischemic stroke patients

(1) Literature Search and Evidence Synthesis

The research team systematically searched relevant Chinese and English databases. English databases included PubMed, Web of Science, EMBASE, and the Cochrane Library. The Chinese databases included China National Knowledge Infrastructure, Wanfang Data, VIP Database, and the Chinese Biomedical Literature Database. Search terms were as follows: “Ischemic stroke or cerebral infarction or cerebral ischemia or embolic strokes or thrombotic stroke or brain infarction,” “medication management or medication adherence or medication compliance or drug adherence or medication-taking behavior or pharmacotherapy management,” and “randomized controlled trial or controlled trial or clinical trial or controlled clinical trial or intervention.” Two researchers independently screened and organized. Moreover, comprehensively analyzed the retrieved literature according to predefined criteria. They extracted the core components and implementation strategies of medication service interventions across different disease stages, thereby providing evidence-based support for developing the intervention program.

(2) Initial Intervention Program Development

Based on the available literature evidence, and considering the disease course, treatment characteristics, and the medication needs of patients and their caregivers at different stages of ischemic stroke, an initial intervention program was developed under the guidance of TIR. The disease trajectory was divided into five phases: Diagnosis, stabilization, discharge preparation, adjustment, and adaptation. Medication management content was designed for each phase, resulting in a draft version of the intervention program.

(3) Expert Consultation

A two-round anonymous Delphi consultation was conducted involving eight senior experts (with at least 10 years of experience) in the fields of cerebrovascular disease, geriatric medicine, pharmacy, and nursing management. These experts evaluated the scientific rigor, feasibility, and clinical applicability of each component of the intervention program. The research team synthesized and analyzed the experts’ feedback, revised the program accordingly, and repeated the process until consensus was reached, resulting in the final intervention program.

(4) Pilot Study and Finalization of the Intervention Program

A pilot study was conducted involving 12 patients with ischemic stroke who were randomly assigned to an intervention group and a control group. Based on the patient feedback and issues identified during implementation, the intervention content and procedures were further refined. The final version of TIR-based medication management program was subsequently established (Supplementary Table 1).

##### 2.2.2.3 Implementation of TIR-based medication management program

Based on the interventions provided to the control group, the intervention group additionally received a TIR-based medication management service program.

(1) Before implementation, the team leader provided training on the medication management program to all team members. To ensure the accuracy and consistency of medication education, the clinical pharmacist conducted rigorous, standardized training and assessment of stroke-related medication knowledge for all intervention providers, ensuring their proficiency in medication knowledge and in applying educational techniques. When novel medications were introduced, the team leader promptly organized additional training and assessment.
(2) The research team conducted qualitative interviews with patients and their primary caregivers to explore their medication management needs. Based on patients’ clinical characteristics, treatment regimens, and stroke risk factor control, stage-specific educational content was developed. Subsequently, this content was integrated into the health education materials, which were delivered through various formats, including video education, QR code-based digital education, slide presentations, group classes, educational handbooks, a family physician platform, and WeChat public account notifications.
(3) The intervention program comprised in-hospital intervention (diagnosis phase, stabilization phase, and discharge preparation phase) and out-of-hospital intervention (implementation phase and adaptation phase). The intervention group received the TIR-based medication management program from admission until 3 months after discharge. (1) In-hospital intervention: During the diagnosis phase, the intervention focused on three core domains: disease cognition (providing a basic, plain-language explanation of stroke), medication safety (initial education on the importance of medications) and psychological support (proactively introduce the ward environment and attending medical staff to reduce unfamiliarity and establish a trusting relationship). In the stabilization phase, emphasis was placed on medication knowledge training for both patients and their caregivers, with one-on-one medication guidance delivered by nurses and clinical pharmacists. During the discharge preparation phase, a personalized and sustainable post-discharge medication management plan was developed to enhance patient acceptance. Training in home-based medication management skills to ensure a seamless transition from hospital to home. (2) During the implementation phase, the focus was on supporting transitional adjustment, providing discharge services, and achieving a three-level linkage model involving hospitals, communities, and families. Community physicians and nurses were notified to take over patient care. Continuity of care was ensured through the global medical community platform and online platforms such as WeChat and DingTalk, thereby establishing effective communication channels among patients, caregivers, and the healthcare team. An electronic medication follow-up record was created for each patient. To ensure effective follow-up, three contact methods were documented: The patient’s own contact information and those of two different close contacts. Both patients and their contacts were informed of the purpose of the follow-up and instructed on how to complete the follow-up record form. The follow-up content consists of three areas: General condition during the home stay, including diet, urination and defecation, sleep, and daily activities; control status of risk factors, such as blood pressure, blood glucose, monitoring frequency, and corresponding results; prescribed medication status, including current types, quantity, dosage, and adverse drug reactions. (3) During the adaptation phase, home-based medication management follow-up was continued, with specialized nurses conducting monthly follow-up visits. For patients with poor medication adherence, the follow-up frequency was increased to weekly visits until regular medication-taking behavior was achieved. After each follow-up, the information was recorded in the patient’s file, enabling the team members to monitor the patient’s home status and implement individualized intervention accordingly. This documentation was reviewed by the clinical pharmacist and the team leader to ensure completeness and accuracy. The pharmacist was responsible for reviewing complex cases and reviewing complex medication-related problems.
(4) Quality assurance. A dedicated DingTalk group was established to facilitate ongoing communication among research team members regarding problems and challenges encountered during program implementation. Monthly team meetings were held at the end of each month to aggregate, exchange, deliberate on, and summarize the issues, enabling continuous program improvement and strengthened quality control.

### 2.3 Questionnaire design

#### 2.3.1 General information questionnaire

This questionnaire was self-developed and primarily collects sociodemographic and clinical data, including patients’ age, sex, educational level, monthly income, type of medical insurance, disorder duration, and other relevant data.

#### 2.3.2 Eight-item Morisky medication adherence scale

The 8-item Morisky Medication Adherence Scale (MMAS-8) was originally developed by Morisky et al. (20) to assess medication-taking behavior. It is widely used as a generic instrument for detecting medication adherence in patients with chronic disorders and remains one of the most commonly employed adherence questionnaires. The scale consists of eight items, with a total score ranging from 0 to 8. Higher scores indicate better adherence: 8 indicates high adherence, 6–7 indicates moderate adherence, and a score below six indicates poor adherence. The Chinese version of the scale was validated by Si et al. (21), who reported a Cronbach’s *α* coefficient of 0.81, a test–retest reliability of 0.95, and a face validity of 1.0, indicating good reliability and validity. In the current study, the pre-survey Cronbach’s α coefficient was 0.74.

#### 2.3.3 Beliefs about medicines questionnaire-specific

Medication beliefs were assessed using the Beliefs about Medicines Questionnaire-Specific (BMQ-Specific), originally developed by Horne et al. (22) and subsequently translated into Chinese and validated by Si et al. (23). The BMQ-Specific consists of two dimensions: Specific-necessity and Specific-concerns. The Specific-necessity dimension assesses patients’ beliefs about their personal need for the prescribed medication. The Specific-concerns dimension assesses patients’ worries about the potential adverse consequences of taking the medication. Each dimension comprises five items, rated on a 5-point Likert scale, with scores for each dimension ranging from 5 to 25. Higher scores indicate stronger beliefs in the corresponding dimension. The scale demonstrated good reliability, with a Cronbach’s *α* coefficient of 0.77, and the test–retest reliability of 0.83.

#### 2.3.4 Medication knowledge questionnaire

Medication knowledge was assessed using a questionnaire adapted from the instrument developed by Ma (24). The original questionnaire, designed for older patients with chronic diseases, has demonstrated good reliability (Cronbach’s *α* = 0.86) and construct validity (0.77) (25). It consists of 15 items, each rated on a 4-point Likert scale. This questionnaire was adapted from the Medication Knowledge Questionnaire to assess medication knowledge in patients with stroke.

### 2.4 Data collection

Data were collected by two trained research assistants. Baseline patient information was obtained from electronic medical records and the General Information Questionnaire upon admission. Medication knowledge was assessed through questionnaire surveys at admission and discharge. MMAS-8 and BMQ scores were collected at four time points: Prior to the intervention, at discharge, and at 1 month and 3 months after discharge.

All questionnaires were distributed and collected on site, and the completeness of responses was verified. Incomplete items were supplemented after confirmation with the patients.

### 2.5 Statistical analysis

All data were entered and analyzed using IBM Statistical Package for the Social Sciences Statistics software (version 23.0). (1) Descriptive statistics were used to present the sociodemographic characteristics (sex, marital status, and occupation) of patients in both groups. (2) Differences between the two groups in medication adherence scores and medication beliefs were compared using independent samples t-tests or Mann–Whitney U tests, as appropriate. (3) Paired samples t-tests were used to compare medication knowledge scores before and after the intervention within each group. (4) Repeated measures analysis of variance (ANOVA) was employed to compare medication adherence scores and medication beliefs between the two groups across four time points (baseline, discharge, 1 month post-discharge, and 3 months post-discharge), and to analyze the group-by-time interaction effect.

## 3 Results

### 3.1 General characteristics

A total of 150 patients were enrolled. Among them, 1 patient died, and 6 were lost to follow-up, resulting in a final sample of 143 patients (70 in the intervention group and 73 in the control group). Statistically non-significant differences were found in baseline general characteristics between the two groups (*p* > 0.05), as illustrated in Table 1.

**Table 1:** Demographics characteristics of patients.

Characteristics		Intervention group (*n* = 70)	Control group (*n* = 73)	Statistic	*p*
Gender, *n* (%)	Men	43 (61.4)	49 (67.1)	0.51	0.48
	Female	27 (38.6)	24 (32.9)		
Age		62.14 ± 11.5	62.2 ± 11.8	−0.05	0.96
Education level	Illiterate	11 (15.7)	13 (17.8)	2.21	0.53
	Primary school or below	22 (31.4)	15 (20.5)		
	Junior high school	23 (32.9)	28 (38.4)		
	Senior high school or above	14 (20.0)	17 (23.3)		
Occupation	Personnel of state organs, party and mass organizations, and enterprises / institutions	1 (1.4)	2 (2.7)	6.11	0.19
	Professional and technical personnel	1 (1.4)	1 (1.4)		
	Commercial and service industry personnel	11(15.7)	8(11.0)		
	Production personnel in agriculture, forestry, fishery, and water conservancy	13 (18.6)	5 (6.8)		
	Other	44 (62.9)	57 (78.1)		
Marital status	Unmarried	0	3 (4.1)	4.09	0.13
	Married	65 (92.9)	65 (89.0)		
	Divorced	0	0		
	Widowed	5 (7.1)	5 (6.9)		
Primary caregiver	Spouse	47 (67.1)	47 (54.8)	3.36	0.50
	Children	19 (27.1)	22 (30.1)		
	Nursing assistant	2 (2.9)	0		
	Parents	0	1 (1.4)		
	None	2 (2.9)	3 (13.7)		
Average monthly household income per capita	<3,000	34 (48.6)	44 (60.3)	2.01	0.37
	3,000–5,000	28 (40.0)	22 (30.1)		
	>5,000	8 (11.4)	7 (9.6)		
Type of medical insurance	Urban employee medical insurance	13 (18.6)	11 (15.1)	6.39	0.10
	Urban and rural resident medical insurance	53 (75.7)	62 (84.9)		
	Commercial insurance	2 (2.9)	0		
	Self-pay	2 (2.9)	0		
Number of comorbid chronic diseases	1	26 (37.1)	35 (47.9)	1.77	0.41
	2	29 (41.4)	24 (32.9)		
	≥ 3	15 (21.4)	14 (19.2)		
Years of illness	1	20 (28.6)	21 (28.8)	1.83	0.61
	>5	13 (18.6)	19 (26.0)		
	>10	20 (28.6)	15 (20.5)		
	>15	17 (24.3)	18 (24.7)		
Daily medication types	1–2	36 (51.4)	44 (60.3)	3.54	0.17
	3–5	29 (41.4)	20 (27.4)		
	≥ 6	5 (7.2)	9 (12.3)		
Medication frequency	Once a day	41 (58.5)	45 (61.7)	0.58	0.75
	Twice a day	13 (18.6)	15 (20.5)		
	Three or more times a day	16 (22.9)	13(17.8)		
Monthly out-of-pocket medication expenses	<100	61 (87.1)	65 (89.00)	2.90	0.23
	100–299	7 (10)	8 (11.0)		
	>300	2 (2.9)	0		
Place of residence	City	30 (42.9)	32 (43.8)	0.014	0.91
	Countryside	40 (57.1)	41 (56.2)		
Importance of self-medication management at home	Yes	50 (71.4)	44 (60.3)	1.97	0.76
	No	20 (28.6)	29 (39.7)		

### 3.2 Medication knowledge at discharge between the two groups

A questionnaire survey was conducted to assess patients’ medication knowledge at Discharge. After receiving medication education, the medication knowledge score in the intervention group (38.21 ± 6.02) was significantly higher compared to that in the control group (34.63 ± 4.98), and the difference was statistically significant (*p* < 0.05), as demonstrated in Table 2.

**Table 2:** Medication knowledge at discharge between the two groups (*X̄* ± s).

Group	Pre-intervention	Post-intervention	*t*	*p*
Intervention group (*n* = 70)	28.64 ± 4.91	38.21 ± 6.02	10.67	<0.001
Control group (*n* = 73)	27.21 ± 2.22	34.63 ± 4.98	11.95	<0.001
Mean difference	1.42 ± 0.80	3.58 ± 0.92	1.99	0.048
*t*	1.87	3.88		
*p*	0.078	<0.001		

### 3.3 Comparison of medication adherence between the two groups at 3 months post-intervention

Prior to the intervention, a statistically non-significant difference was found in medication adherence scores between the two groups (*p* > 0.05). After implementation of the management program, the medication adherence score in the intervention group was significantly higher compared to that in the control group (*p* < 0.05). The results of repeated measures ANOVA indicated statistically significant differences in medication adherence scores between the two groups in patients with stroke in terms of between-group effects, time effects, and group-by-time interaction effects, as depicted in Table 3.

**Table 3:** Repeated measures ANOVA of medication adherence scores in two groups of patients (*X̄* ± s).

Group	Pre-intervention	Post-intervention	1 month post-intervention	3 month post-intervention	Between-subjects effect *F(p)*	Time effect *F(p)*	Interaction effect *F(p)*
Intervention group (*n* = 70)	7.06 ± 1.28	7.89 ± 0.37	7.69 ± 0.73	7.56 ± 0.83	17.45 (<0.001)	43.27 (<0.001)	3.28 (0.023)
Control group (*n* = 73)	6.73 ± 0.91	7.60 ± 0.72	7.30 ± 1.13	6.71 ± 1.57			
*t*	1.74	3.86	2.44	4.01			
*p*	0.083	<0.001	0.016	<0.001			

### 3.4 Comparison of medication beliefs between the two groups at 3 months post-intervention

Prior to the intervention, statistically non-significant differences were identified between the two groups in the scores for the specific-necessity dimension and the specific-concerns dimension of medication beliefs (*p* > 0.05). After 3 months of implementing the management program, the intervention group demonstrated significantly higher scores in the specific-necessity dimension and significantly lower scores on the specific-concerns dimension than the control group, with statistically significant differences (*p* < 0.05). Repeated measures ANOVA demonstrated significant between‑group and time effects for both necessity and concerns scores. The group‑by‑time interaction was significant for the necessity dimension (*p* < 0.05) but not for the concerns dimension (*p* > 0.05). Details are presented in Tables 4, 5.

**Table 4:** Repeated measures ANOVA of medication specific-necessity dimension in two groups of patients (*X̄* ± s).

Group	Pre-intervention	Post-intervention	1 month post-intervention	3 month post-intervention	Between-subjects effect *F(p)*	Time effect *F(p)*	Interaction effect *F(p)*
Intervention group (*n* = 70)	14.64 ± 3.89	22.67 ± 3.26	21.91 ± 3.71	21.98 ± 3.65	42.33 (<0.001)	106.18 (<0.001)	12.37 (0.003)
Control group (*n* = 73)	14.11 ± 4.24	20.30 ± 2.54	18.90 ± 2.23	17.47 ± 2.84			
*t*	0.78	4.86	5.89	8.23			
*p*	0.435	<0.001	<0.001	<0.001			

**Table 5:** Repeated measures ANOVA of medication specific-concerns dimension in two groups of patients (*X̄* ± s).

Group	Pre-intervention	Post-intervention	1 month post-intervention	3 month post-intervention	Between-subjects effect *F(P)*	Time effect *F(P)*	Interaction effect *F(P)*
Intervention group (*n* = 70)	13.41 ± 3.43	10.76 ± 3.40	8.41 ± 3.65	7.54 ± 3.65	18.77 (<0.001)	89.99 (<0.001)	1.31 (0.275)
Control group (*n* = 73)	14.19 ± 2.71	12.34 ± 2.20	10.59 ± 3.05	9.56 ± 2.71			
*t*	−1.51	−3.32	−3.88	−3.76			
*p*	0.05	0.01	<0.001	<0.001			

## 4 Discussion

Several considerations informed our decision to restrict the study population to patients with ischemic stroke. First, ischemic stroke is the most common subtype, accounting for approximately 77.8% of all stroke cases. By focusing on this predominant subtype, our findings achieve broader clinical representativeness and applicability. Second, a single-subtype focus enables more precise alignment with the TIR theoretical framework. Based on TIR, this study developed a continuous, multidisciplinary medication management model covering the five stages of “diagnosis, stabilization, discharge preparation, adjustment, and adaptation.” It evaluated its effects on medication adherence and medication beliefs. The results demonstrated that patients in the intervention group had significantly better medication knowledge, medication adherence, and medication beliefs than those in the control group, and that the intervention effects persisted for 3 months following discharge. The findings are discussed below.

### 4.1 TIR-based medication management program effectively improves patients’ medication knowledge

The results of this study demonstrated that at discharge, patients in the intervention group had significantly higher medication knowledge scores (38.21 ± 6.02) compared with those in the control group (34.62 ± 4.98; *p* < 0.05), indicating that stage-specific medication education based on TIR promotes better medication knowledge acquisition compared to conventional care. This finding may be attributed to the phased, progressive, and systematic design of the medication management program. TIR emphasizes the provision of targeted support tailored to patients’ changing needs across different stages of the disorder trajectory.

During the acute phase (diagnosis phase), patients experience a heavy cognitive load. Consequently, the intervention focuses on medication safety and emotional support rather than knowledge transmission, thereby establishing a solid foundation for subsequent health education. As the patient’s condition stabilizes (stabilization and discharge preparation phases), their receptiveness to information improves. At this stage, systematic and personalized education is delivered through one-on-one intensive guidance, QR code video education, medication list development, and scenario-based simulation training. This approach aligns with patients’ readiness to learn, ensures both the depth and breadth of knowledge transfer, and improves the efficiency of information absorption. This is aligned with the findings of Chu et al. (26).

The adoption of a multidisciplinary team-based working model ensures the accuracy and consistency of information. Clinical pharmacists, physicians, and nurses collaborate to provide explanations from several dimensions, such as drug mechanisms, treatment goals, and daily management, thereby preventing potential biases inherent in single-source information, reinforcing the authority of the knowledge delivered, and enhancing patient trust. Recent guidelines support tailored risk factor management and multidisciplinary, team-based care to enhance secondary stroke prevention (27). Notably, the difference in medication knowledge between the two groups at discharge did not immediately translate into differences in medication adherence, indicating that knowledge acquisition is necessary for behavioral change. The transition from knowledge to action depends on belief formation and sustained behavioral reinforcement, thereby highlighting the need for follow-up and support during the later stages of our medication service program.

### 4.2 TIR-based medication management program optimizes patients’ medication beliefs

Repeated measures ANOVA revealed significant between-group differences in medication belief scores at discharge, 1 month post-discharge, and 3 months post-discharge. Patients in the intervention group scored significantly higher on the specific-necessity dimension and significantly lower on the specific-concerns dimension compared with those in the control group (*p* < 0.05). These findings indicate that a TIR-based medication management program not only enhances patients’ recognition of the value of secondary preventive medications but also effectively alleviates their concerns and worries about the medications. Medication beliefs are considered a modifiable factor that significantly influences medication adherence (28). The formation of medication beliefs is a dynamic process that needs continuous information input and cognitive restructuring (13). Patients with ischemic stroke typically require lifelong pharmacotherapy. Over time, patients’ perceived benefits of treatment may diminish, leading to an increased sense of disease burden and, consequently, cognitive biases.

The medication management program in this study effectively optimized patients’ medication beliefs through three main strategies. First, it reinforced the perceived necessity of medication through several channels. Throughout the multi-stage intervention, team members continuously emphasized to patients and their caregivers the importance of secondary preventive medications for ischemic stroke and the critical role of medication adherence in preventing stroke recurrence, thereby explicitly linking medication-taking to long-term health outcomes. This enhanced patients’ perception of the necessity of medication and, consequently, strengthened their motivation to adhere to it. This finding suggests that enhancing the perception of disease severity and treatment necessity is central to improving medication beliefs (29). Second, proactive education was used to address medication concerns prospectively. Rather than avoiding discussions of potential adverse drug reactions, the intervention proactively educated patients during the stabilization and discharge preparation phases, informing them how to identify common side effects and manage them. When patients reported problems, immediate professional solutions were provided. This open, supportive communication effectively reduced patients’ worries and uncertainty, thereby diminishing their concerns. This finding is aligned with the meta-analysis by Sheils et al. (29), demonstrating that targeted interventions can significantly reduce patients’ concerns about medications. Finally, continuous social support reinforcement is provided. Emotional support and information from family members, healthcare teams, and the community can enhance patients’ perceived treatment benefits and alleviate their sense of disease burden, thereby effectively preventing the development of cognitive biases. The combined effect of sustained informational and social support can improve patients’ attitudes toward medication. The multi-stage, multi-agent medication management model employed in this study serves as a practical embodiment of this concept.

### 4.3 TIR-based medication management program significantly improves patients’ medication adherence

Repeated measures ANOVA revealed significant between-group differences in medication adherence scores at discharge, 1 month post-discharge, and 3 months post-discharge (*p* < 0.05), with the intervention group consistently achieving higher scores compared to the control group. This finding is consistent with previous studies on nursing interventions based on TIR (17, 18), suggesting that a stage-specific, needs-oriented intervention model possesses unique advantages in promoting behavioral change among patients with chronic disorders.

The sustained effect of the intervention can be attributed to the precise alignment of the program with the adherence barriers patients face at different stages. The program was developed from the perspective of patients’ needs, progressing step by step through the five stages of TIR in a coherent, sequential manner. During the diagnosis phase, the focus was on building trust and ensuring medication safety. Interventions such as “directly observed therapy” (to ensure that medication is taken by mouth) were implemented to initially establish standardized medication-taking behavior, thereby laying a basis for subsequent interventions. During the stabilization phase, the measurement focused on systematic knowledge education and belief guidance. Multidisciplinary team-based intensive instruction, medication belief assessment, and secondary prevention knowledge dissemination were provided to strengthen patients’ understanding of their disease and medication while initially reshaping their attitudes toward treatment, thereby motivating them to take their medications willingly. The discharge preparation phase served as a critical period for behavioral transition. The primary challenge faced by patients during this phase was the transition from the hospital to the home environment. Through individualized medication plans, skills training, and scenario-based simulations, abstract knowledge was converted into actionable self-management abilities, ensuring correct medication administration and completing the essential shift from knowledge to action (30). The adjustment and adaptation phases provided continuous follow-up and support, creating an external support system. The first 3 months after discharge represent a critical period for declining adherence. As symptoms alleviate and daily life returns to normal, issues such as forgetting to take medications and concerns about side effects gradually become significant (10). Our telephone follow-up during this phase actively identified and addressed barriers, such as simplifying regimens, strengthening reminders, and explaining the manageability of side effects, thereby halting the decline in adherence. This approach helped cement medication-taking as a stable habit, promoting sustained long-term adherence. A systematic review by Dalli et al. (13) confirmed that interventions based on TIR can ultimately improve patient care outcomes by addressing the evolving needs of caregivers at different stages. Moreover, research by D’Alton et al. (31) highlighted that patients with less severe disability, who may feel less reliant on medications, constitute a high-risk group for poor adherence, indicating that post-discharge continuous support is critical to prevent adherence decline. Therefore, by addressing the dynamic needs of patients across varying stages of the disorder, our medication management program achieved an effective transformation from “knowledge” to “belief” and ultimately to “action.”

### 4.4 Limitations

This study has several limitations. First, the use of convenience sampling and non-random allocation by hospital floor constitutes a quasi-experimental design rather than a true randomized controlled trial. Although baseline characteristics were comparable between groups, we cannot entirely exclude the possibility of unmeasured confounding. Future studies employing rigorous randomized controlled designs are needed to confirm our findings. Second, the sample was derived from a single center, potentially introducing selection bias. Third, the intervention effects were only followed up to 3 months post-discharge, leaving the sustainability of long-term adherence unclear. Fourth, some of the online interventions implemented during the out-of-hospital phase could not capture patients’ and caregivers’ non-verbal cues, which may have compromised assessment accuracy and precluded timely feedback.

## 5 Conclusion

TIR-based medication adherence program for patients with ischemic stroke employs dynamic, stage-specific, and supportive interventions. Through continuous medication education, reinforcement of social support, and cognitive restructuring, the program significantly improved medication adherence, optimized medication beliefs, reduced patients’ perceived disease burden, and prevented adherence breakdown caused by cognitive biases. It also enhanced engagement from caregivers and the community, enabling collaborative management that further strengthened patients’ medication beliefs and adherence.

## Data Availability

The raw data supporting the conclusions of this article will be made available by the authors, without undue reservation.
