# Supplementary material for: Construction and application of a medication adherence management program based on TIR for patients with ischemic stroke
Source: Front Public Health. 2026 Jul 24;14:1869629. doi: 10.3389/fpubh.2026.1869629 (PMC13447297; doi:10.3389/fpubh.2026.1869629)
Supplement: Supplementary file 1 [file Table_1.DOCX]

Supplementary Material

# 1 Supplementary Tables

# Supplementary Table 1 Drug Service Management Intervention Plan for Ischemic Stroke Patients Based on the theory of Timing It Right

| Intervention Stage | Intervention Time | Key Needs of Patients and Caregivers | Intervention Content |
| --- | --- | --- | --- |
| Diagnostic Period (from disease onset to confirmed diagnosis) | Acute Admission Stage | **Disease Cognition** | Utilize a combination of videos, group lectures, and bedside explanations to educate patients and caregivers on:  1. Definition and etiology of ischemic stroke;  2. Common clinical manifestations and identification;  3. Main diagnostic methods and treatment principles;  4. Disease prognosis and predictive factors. |
|  |  | **Medication Safety** | 1. Standardize the medication administration process, implement "medication delivery to the bedside", and reinforce the importance of timely medication intake;  2. When dispensing medications, briefly explain the drug name, purpose, and key precautions to help patients and caregivers become familiar with their therapeutic drugs;  3. Strengthen protocol adherence. For special circumstances such as leaving the ward for examinations, use bedside reminder signs to prevent missed doses;  4. Encourage family participation to enhance medication safety. |
|  |  | **Psychological Support** | 1. Proactively introduce the ward environment and attending medical staff to reduce unfamiliarity and establish a trusting relationship;  2. Assess emotional status and provide targeted psychological counseling to alleviate tension and anxiety;  3. Promptly address and meet the psychological needs of patients. |
| Stable Period (from definite diagnosis to stable condition ) | During Inpatient Treatment | **Medication Management** | 1. Systematically assess patients’ medication beliefs, cognitive function, and social support systems to identify potential barriers to adherence;  2. Nurses provide one-on-one intensive guidance during drug dispensing, covering drug name, purpose, dosage, timing, route, and potential adverse drug reactions;  3. Provide a convenient drug information QR code for patients and caregivers to access;  4. The head nurse and clinical pharmacist conduct daily checks on education implementation, reinforcing unmastered content until proficiency is achieved;  5. Closely monitor and promptly address any adverse drug reactions. |
|  |  | **Psychological Support** | Timely explain to patients and caregivers the current disease stage, expected prognosis, and key nursing points. Encourage a positive attitude and enhance confidence in rehabilitation. |
|  |  | **Awareness of Secondary Prevention** | Through a combination of videos, group lectures, and bedside guidance, introduce:  1. The concept and importance of secondary prevention;  2. Major risk factors for stroke;  3. The vital role of standardized medication adherence in treatment. |
| Discharge Preparation Period (from stable condition to completion of discharge procedures) | Adjustment Period (1-3 Months After Discharge) | **Medication Management** | 1. Develop a sustainable, personalized medication plan: Incorporate the patient's perspectives, preferences, daily medication management habits, lifestyle rhythm, and family environment to jointly formulate a feasible medication plan;  2. Provide an individualized medication list: Clearly list drug names, dosages, administration instructions, treatment duration, and key precautions;  3. Guide the establishment of a medication and health monitoring diary: Record medication intake, adverse reactions, and indicators such as blood pressure / blood glucose;  4. Conduct self-management skills training: Include dosage calculation, pill organization, storage methods, handling missed doses, proper drug use, medication reminder setup, and drug procurement channels;  5. Conduct medication scenario simulations: Simulate situations such as medication purchase, administration, storage, and adverse reaction monitoring to promptly identify and correct issues problems. |
|  |  | **Discharge Preparation** | 1. Explain the discharge process in detail and jointly develop a practical discharge plan;  2. Introduce "Internet +" services such as online consultation, follow-up appointment scheduling, and home care options, establishing smooth communication channels. |
|  |  | Referral Linkage | Assess the patient's post-discharge community support conditions, contact the community health service center in advance, and ensure seamless transition of medical and nursing services. |
| Adjustment Period (from discharge to 1 month) | Discharge Transition Stage | **Continuous Medication Management** | 1. Conduct a telephone follow-up 72 hours after discharge, followed by weekly telephone follow-ups every Monday to monitor the patient's condition and address questions;  2. Schedule and assist in completing an in-person follow-up visit one month after discharge for intensive guidance;  3. Provide convenient online and telephone medication consultation services;  4. For patients with poor medication adherence or special difficulties, conduct home visits after assessment. |
|  |  | **Psychological and Social Support** | 1. Guide the effective utilization of community resources;  2. Provide timely doubt-resolution and emotional support through WeChat, telephone, and other communication methods. |
| Adaptation Period(1to3 months after discharge) | Home Consolidation Stage | Continuous Medication Management | 1. The nurse conducts a telephone follow-up on each month to assess the patient’s home medication management;  2. For patients who become lax in medication adherence or those whose vigilance decreases due to asymptomatic conditions, the clinical pharmacist conducts telephone communication. The strategy shifts from supervision to empowerment, encouraging medication diary-keeping and attention to health indicators;  3. For patients with poor medication adherence, conduct home visits when necessary;  4. Send WeChat notifications on secondary prevention knowledge every two weeks;  5. Organize online or offline health lectures on the 10th of each month. |
|  |  | **Social Reintegration and Health Promotion** | 1. Provide positive feedback and praise to patients with good medication compliance to reinforce positive behaviors;  2. Encourage active use of community health services, standardized chronic disease management, gradual adjustment of daily routines, and social reintegration;  3. For patients and caregivers experiencing negative emotions, provide timely guidance to help them adjust their mindset and pace of life. |
